# A Challenging Diagnosis of Hemophagocytic Lymphohistiocytosis with Unusual Pulmonary Manifestations: A Case Report

**DOI:** 10.2174/0118743064414458251015063933

**Published:** 2025-11-06

**Authors:** Claudia Carducci, Paola Parronchi, Emilio Portaccio, Maria Pia Amato, Luisa Pastó, Filippo Bartalesi, Gioia Di Stefano, Valeria Pasini, Camilla Eva Comin, Claudia Ravaglia, Venerino Poletti, Federico Lavorini, Sara Tomassetti

**Affiliations:** 1 Pulmonology and Thoraco-pulmonary Pathophysiology Unit, Department of Experimental and Clinical Medicine, Azienda Ospedaliero-Universitaria Careggi, Florence, Italy.; 2 Immunology and Cell Therapy Unit, Department of Experimental and Clinical Medicine, Azienda Ospedaliero-Universitaria Careggi, Florence, Italy; 3 Department of Neuroscience, Psychology, Drug Research and Child Health, Azienda Ospedaliero-Universitaria Careggi, Florence, Italy; 4 Department of Medical and Surgical Critical Area, Infectious Diseases Clinic, Azienda Ospedaliero-Universitaria Careggi, Florence, Italy; 5 Pathological Anatomy Section, Azienda Ospedaliero-Universitaria Careggi, Florence, Italy; 6 Department of Diseases of the Thorax, Ospedale G.B. Morgagni - L.Pierantoni, Forli, Italy; 7 Interventional Pneumology, Department of Experimental and Clinical Medicine, Azienda Ospedaliero-Universitaria Careggi, Florence, Italy

**Keywords:** Hemophagocytic lymphohistiocytosis, HLH, Respiratory distress, Perforin, Lung biopsy, Respiratory failure

## Abstract

**Introduction:**

Hemophagocytic Lymphohistiocytosis (HLH) is a rare, aggressive, and life-threatening disorder characterized by sustained but ineffective immune system activation that leads to severe and systemic hyperinflammation. It may occur as a genetic or sporadic condition, often triggered by an infection. The multifaceted pathogenesis results in a wide range of non-specific symptoms, signs, and laboratory findings that challenge its recognition. The pulmonary involvement is underdiagnosed and may manifest as pneumonia, which can lead to respiratory failure. Despite the great improvement achieved in terms of survival, a considerable proportion of patients with HLH still die from progressive disease.

**Case Presentation:**

We discuss the case of a unique form of respiratory distress and multiorgan failure with inconclusive radiological and lung biopsy investigations. The patient was finally diagnosed, by genetic analysis, with HLH, and promptly treated as per HLH-94 treatment protocol.

**Conclusion:**

This case report aims to emphasize that clinical, laboratory, instrumental, and even pathological findings in HLH might not be unequivocal; nonetheless, a rapid diagnosis and treatment are mandatory, given the high mortality of the disease.

## 1 INTRODUCTION

The hemophagocytic lymphohistiocytosis (HLH) represents the archetype of hyperinflammation syndrome [1]. It is a rare, aggressive, and life-threatening disorder, characterized by excessive inflammation and tissue destruction.

HLH can occur as a familial (FHL) or sporadic disorder. Genetic defects play a major role in childhood HLH but are increasingly found in adult cases [2]. Due to the varying penetrance of genetic mutations, less pathogenic gene variants might promote the onset of HLH-like forms, where hemophagocytosis occurs subsequently or alongside hematologic malignancies, particularly non-Hodgkin lymphomas, rheumatologic conditions, autoinflammatory and autoimmune disorders, or other inherited immunodeficiency diseases. In these cases, the very first onset of disease might be triggered by infectious organisms, such as the Epstein-Barr virus (EBV) [3]. Therefore, HLH can be considered as a dynamic continuum of inflammatory conditions, where the risk of developing the syndrome is the result of the subtle balance between a predisposed genotype and environmental factors [4].

The pathogenesis of the familial HLH is distinctive. In the FHL, the normal perforin-dependent cytotoxicity of damaged, stressed, infected, or transformed host cells by natural killer (NK) and cytotoxic T lymphocytes (CTLs) fails. The target cells survive, become hyperreactive, and start secreting excessive amounts of cytokines, especially interferon (IFN)- gamma, tumor necrosis factor (TNF)-alpha, interleukin (IL) 1, 4, 6, 8, 10, and 18, causing a cytokine storm with multiorgan failure and eventually death [5-7].

The diagnosis of HLH syndrome is based on a compatible clinical presentation in the setting of elevated inflammatory markers and follows the HLH-2004 criteria [8]. Although the HLH-2004 criteria have some limitations because they were developed in the pediatric context, they are regularly adopted in clinical settings [4].

The most established treatment protocols for primary HLH, the HLH-94 and subsequent HLH-2004 protocols, are based on the use of etoposide, dexamethasone, and cyclosporine [1]. Allogeneic hematopoietic stem cell transplantation (HSCT) is currently the only option for long-term cure in primary HLH [9].

The pathophysiology of secondary HLH is not yet fully understood, and best practices for diagnosis and treatment of the various forms of secondary HLH deserve further attention [1].

Despite recent improvements in survival, around 40% of patients with HLH still die from refractory disease or toxicity [4].

Recent advances in computational medicine, such as adaptive ensemble deep learning frameworks [10], may in the future complement clinical expertise and aid earlier recognition of HLH, particularly when triggered by infections.

Our case is a unique and challenging pulmonary presentation of a complicated FHL that shows how lung involvement remains underdiagnosed, although it can lead to respiratory failure and potentially death [11]. It also demonstrates that innovative approaches, integrated in a multidisciplinary setting [12], are mandatory given the high complexity of the disease and its high mortality.

## 2 CASE PRESENTATION

We discuss the case of a 50-year-old non-smoker woman. From her past medical history, we report bronchial asthma, treated with low-dose inhaled corticosteroid, and multiple sclerosis (MS) with atypical/vasculitic characteristics, treated with Natalizumab.

**Fig. (1):**
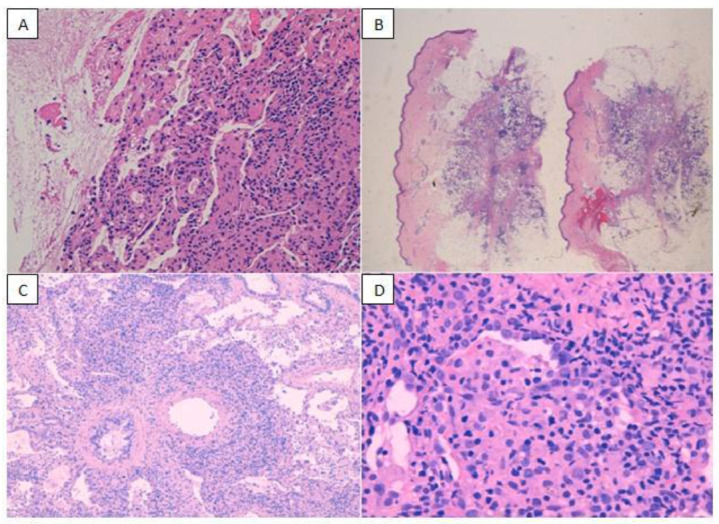
**(A)** Microscopic appearance of lung parenchyma stained with hematoxylin eosin (HE) showing peribronchiolar lympho-histiocytic infiltrate; **(B)** Dermo-hypodermic layer of skin from the lower limb stained with HE that demonstrates chronic inflammatory infiltrate lymphocytes; **(C)** Lung parenchyma stained with HE showing peribronchovascular infiltrate consisting of lymphocytes and plasma cells; **(D)** Detail of lymphocytes at greater magnification.

At the age of 39, she developed lung infiltrates, severe cytopenia, and skin nodules. Biologic treatment was thus stopped, and bone marrow examination, along with multiple lung and skin biopsies (Fig. **1A-D**), were performed. The diagnosis of a lymphoproliferative EBV-related disorder was made, but later on was defined as peripheral T-cell lymphoma not otherwise specified (PTCL-NOS) with pulmonary and cutaneous involvement.

Accordingly, the patient was subjected to chemotherapy and an autologous bone marrow transplant, finally achieving complete remission.

After a long healthy period without any additional therapy, at the age of 47, neurological signs and new brain lesions, not properly explained by MS, appeared again. Thus, a biopsy of the nervous tissue was performed, revealing a perivascular lymphoid infiltrate with low proliferation and oligoclonal T cells, associated with isolated histiocytes. Taken together, these findings excluded a brain localization of a lymphomatous malignancy and led to the diagnosis of a chronic demyelinating inflammatory disease compatible with atypical multiple sclerosis/chronic lymphocytic inflammation with pontine perivascular enhancement responsive to steroids (CLIPPERS)-like lesions. A second-line therapy for multiple sclerosis was, thus, started.

**Fig. (2):**
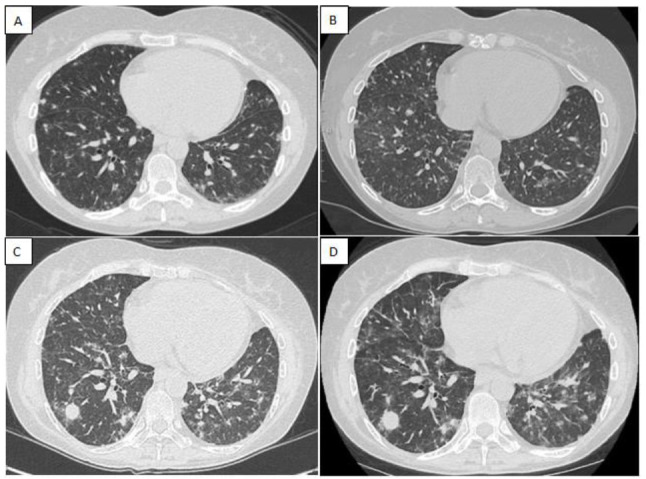
**(A)** Lung CT shows bilateral parenchymal nodules with predominantly peribronchovascular distribution with slightly blurred margins, surrounded by ground glass areas; **(B)** Slight increase in micro nodularities and ground glass findings; **(C)** Appearance of major parenchymal consolidations with air crescent sign in the lateral segment of the right lower lobe; **(D)** Dimensional increase of the previously described consolidation with simultaneous worsening of the remaining findings.

Two years later, nodular infiltrates on the lower limbs appeared again, and after six months, the patient accessed our hub center for increasing exertional dyspnea and persistent fatigue. The new thorax computed tomography (CT) (Fig. **2A**) revealed nonspecific micro and macro pulmonary nodularities with peripheral ground glass, while the positron emission tomography (PET) scan showed pathological, high glucose metabolism tissue in the lungs.

As a result, a recurrence of the previously defined lymphoproliferative disease was suspected, but other causes, such as sarcoidosis or opportunistic infections, could not be excluded with certainty. Autoimmunity tests were negative, while microbiological research and chitotriosidase dosage were inconclusive, and the patient could not undergo any specific therapy.

After a few months, clinical and related CT (Fig. **2B**) and PET findings worsened. Laboratory tests exhibited profound pancytopenia. Consequently, a new bone marrow biopsy was performed and demonstrated the presence of lymphocytes and histiocytic-macrophagic elements, with no evidence of lymphoproliferative process in progress.

Given the clinical and instrumental (Fig. **2C** and **D**) worsening, two pulmonary cryobiopsies were performed in 3 months: both confirmed an atypical nonspecific peribronchovascular lympho-histiocytic infiltrate. The progression of lung involvement led to partial respiratory failure requiring oxygen therapy.

Nevertheless, a diagnosis of certainty failed again, and the patient was discharged, in partially clinically stable conditions, without any specific treatment except for her chronic therapy, prophylaxis against opportunistic infections, and oxygen therapy.

Shortly after, the patient was once more hospitalized as fever (>38.5°C), gait abnormality, dysmetria, worsening of ataxia, and left limb hyposthenia were observed.

## 3 DISCUSSION

In view of a possible unifying disease based on multiorgan involvement, clinical features, histological, and laboratory findings, a complete revision of possible differential diagnoses was considered:

### 3.1 Primary Immunodeficiency with Lymphoproliferation

- CTLA4 haploinsufficiency: The research for the CTLA4 mutation resulted in negative.
- LRBA deficiency: It was considered unlikely, as it is generally characterized by more aggressive pictures.
- Autoimmune lymphoproliferative syndrome (ALPS): CD3+CD4-CD8-αβ+ double-negative lymphocytes were within the normal range.
- Chronic Active Epstein–Barr Virus Disease (CA-EBV): It was unlikely, and EBV-DNA was repeatedly negative.

### 3.2 Intravascular T Cell Lymphoma Recurrence

It was ruled out through review of bone marrow biopsies.

### 3.3 HLH, as Laboratory and Instrumental Investigations Together with Clinical Findings, were Highly Suspected of Hemophagocytosis

- Non-infectious fever (broken with corticosteroids).
- Modest splenomegaly (maximum diameter on the long axis 13 cm) with echo-structure alterations due to hemocateresis.
- Trilinear cytopenia (Hb 8.1 g/dl, WBC 1.14 × 10^9^/L, PLT 82× 10^9^/L).
- Increased triglycerides (286 mg/dl) and low fibrinogen values (120 mg/dl).
- Increased ferritin (7970 ng/ml).
- Modest hemophagocytosis in bone marrow biopsy (at second revision) with definite exclusion of clonal T cell proliferation and EBV-DNA expression.
- Defective expression of perforin by flow cytometry.
- Three missense mutations of the gene *PRF1*:

- [3G>A]+[=], p. [Met1lle]+[=]

- [272C>T]+[=], p. [Ala91Val]+[=]

- [1153C>T]+[-], p. [Arg385Trpl]+[=]

The first two were already described as pathogenic. The familiarity was not tested because the patient was adopted.

It is worth noting that, although hematological malignancies and viral infections are well-recognized triggers of HLH, the patient had already exhibited features of the disease at the time of her initial diagnosis of lymphoma with pulmonary and cutaneous involvement. Notably, this was also preceded by the atypical central nervous system (CNS) inflammation, which was subsequently reinterpreted as a manifestation of the HLH spectrum itself.

### 3.4 Chronic Lymphocytic Inflammation with Pontine Perivascular Enhancement Responsive to Steroids (**CLIPPERS**)

The above-described CNS involvement, initially misclassified as atypical multiple sclerosis, mimicked a CLIPPERS syndrome. However, clinical recurrence after steroid withdrawal, the absence of typical gadolinium-enhancing patterns on brain MRI, and the emergence of systemic signs of HLH pointed to a CLIPPERS-like central nervous system manifestation of HLH.

High-dose steroids (dexamethasone 10 mg/m^2^ once a day) and immunosuppressive drugs (etoposide 100 mg/m^2^ once a day) were started with rapid neurological improvement, withdrawal of oxygen therapy, and fever regression.

One year later, the patient was again hospitalized for radiological and symptomatological exacerbation of the central nervous system disease, including the onset of diplopia, and treated with high-dose steroids. The respiratory condition remained stable.

The patient is currently undergoing bone marrow transplant evaluation.

## CONCLUSION

Hemophagocytic lymphohistiocytosis is a life-threatening condition associated with multiple organ dysfunction, including respiratory distress, whose early diagnosis is critical for treatment.

In this case report, we aim to demonstrate that lung CT and PET findings are absolutely nonspecific, rapidly change over time, and may mimic other conditions.

Lung biopsy, although repeated, might not be pathognomonic for hemophagocytosis or might even result in inconclusive results despite specific staining and molecular investigations. However, since the course of disease is unpredictable, clinical and laboratory re-evaluations must be performed. Looking ahead, computational medicine assumes relevance in late-onset primary forms of HLH, where clinical features are often diverse and inconsistently collected, lacking the rigor, uniformity, or structured approach typically required for a reliable diagnostic framework [13].

Ultimately, our case exemplifies why innovative approaches, integrated in a multidisciplinary setting, are urgently needed and might be essential for rapid diagnosis and prompt treatment of hemophagocytic lymphohistio-cytosis.

## Data Availability

The history, laboratory exams, histological and radiological pictures which come from the "Università degli Studi di Firenze" and "AOUC Careggi" archives, obtained with permission of the patient from corresponding author [C.C].

## LIST OF ABBREVIATIONS

HLH: Hemophagocytic Lymphohistiocytosis
FHL: Familial Hemophagocytic Lymphohistiocytosis
EBV: Epstein-Barr Virus
NK: Natural Killer
CTL: Cytotoxic T Lymphocytes
IFN: Interferon
TNF: Tumor Necrosis Factor
IL: Interleukin
HSCT: Hematopoietic Stem Cell Transplantation
PTCL- NOS: Peripheral T-Cell Lymphoma Not Otherwise Specified
CLIPPERS: Chronic Lymphocytic Inflammation with Pontine Perivascular Enhancement Responsive to Steroids
CTLA4: Cytotoxic T-Lymphocyte Associated protein 4
LRBA: Lipopolysaccharide-responsive beige-like anchor
ALPS: Autoimmune lymphoproliferative syndrome
CA-EBV: Chronic Active Epstein–Barr Virus Disease

## STANDARDS OF REPORTING

CARE guidelines were followed.
